# Supplementary material for: A lung-on-chip model of early Mycobacterium tuberculosis infection reveals an essential role for alveolar epithelial cells in controlling bacterial growth
Source: eLife. 2020 Nov 24;9:e59961. doi: 10.7554/eLife.59961 (PMC7735758; doi:10.7554/eLife.59961)
Supplement: Transparent reporting form [file elife-59961-transrepform.docx]

***eLife’s* transparent reporting form**

We encourage authors to provide detailed information *within their submission* to facilitate the interpretation and replication of experiments. Authors can upload supporting documentation to indicate the use of appropriate reporting guidelines for health-related research (see [EQUATOR Network](http://www.equator-network.org/%20)), life science research (see the [BioSharing Information Resource](https://biosharing.org/)), or the [ARRIVE guidelines](http://www.plosbiology.org/article/info:doi/10.1371/journal.pbio.1000412) for reporting work involving animal research. Where applicable, authors should refer to any relevant reporting standards documents in this form.

If you have any questions, please consult our Journal Policies and/or contact us: [editorial@elifesciences.org](mailto:editorial@elifesciences.org).

**Sample-size estimation**

- You should state whether an appropriate sample size was computed when the study was being designed
- You should state the statistical method of sample size computation and any required assumptions
- If no explicit power analysis was used, you should describe how you decided what sample (replicate) size (number) to use

Please outline where this information can be found within the submission (e.g., sections or figure legends), or explain why this information doesn’t apply to your submission:

For data reporting intracellular bacterial growth on lung-on-chip devices, every microcolony within 16-25 fields of view on any given device were analysed, corresponding to most of the surface area of the chip. We also show in Figure 2 – figure supplement 4 that growth outcomes are consistent throughout the length of the chip. Innocula of between 200-800 bacteria per chip generated sufficient data (n>=55 for macrophage infection and n>=25 for alveolar epithelial cell (AT) infection for statistical comparisons between AT and macrophage populations. Data from WT were obtained from two LoCs to demonstrate the reproducibility of the system.

Analysis of confocal images of pro-SPC+ lamellar bodies in normal and deficient surfactant level ATs in cell culture: n=3 image volumes for each population were acquired and n=2 volumes were analysed to identify the number, intensity, and volume of lamellar bodies.

Analysis of confocal images of pro-SPC+ lamellar bodies in the epithelial layer of DS and NS LoCs: n=5 (DS) and n=6 (NS) volumes were acquired and analysed to identify the number and volume of lamellar bodies and the intensity of proSPC immunofluorescence.

Analysis of AT infection in a single-cell suspension of dissociated mouse lung: approximately 2/3rd of the volume of the lung was visually inspected for Mtb infection of type II ATs. The remaining portion of lung was used other analyses and colony forming unit counts. A similar fraction of the lung of another mouse were inspected for Mtb infection of type I ATs.

**Replicates**

- You should report how often each experiment was performed
- You should include a definition of biological versus technical replication
- The data obtained should be provided and sufficient information should be provided to indicate the number of independent biological and/or technical replicates
- If you encountered any outliers, you should describe how these were handled
- Criteria for exclusion/inclusion of data should be clearly stated
- High-throughput sequence data should be uploaded before submission, with a private link for reviewers provided (these are available from both GEO and ArrayExpress)

Please outline where this information can be found within the submission (e.g., sections or figure legends), or explain why this information doesn’t apply to your submission:

For data reporting single cell intracellular bacterial growth on lung-on-chip devices, a vast majority of the surface area (16-25 fields of view) of the chip was imaged and every microcolony within these fields of view were analysed (either macrophage or AT infection). In addition, experiments with WT Mtb in normal and deficient surfactant conditions were repeated atleast twice to demonstrate reproducibility between chips. All information for the number of microcolonies analysed for each experiment are included in the Figure Legends and in Table 2.

**Statistical reporting**

- Statistical analysis methods should be described and justified
- Raw data should be presented in figures whenever informative to do so (typically when N per group is less than 10)
- For each experiment, you should identify the statistical tests used, exact values of N, definitions of center, methods of multiple test correction, and dispersion and precision measures (e.g., mean, median, SD, SEM, confidence intervals; and, for the major substantive results, a measure of effect size (e.g., Pearson's r, Cohen's d)
- Report exact p-values wherever possible alongside the summary statistics and 95% confidence intervals. These should be reported for all key questions and not only when the p-value is less than 0.05.

Please outline where this information can be found within the submission (e.g., sections or figure legends), or explain why this information doesn’t apply to your submission:

All information regarding the statistical tests used, the exact N values, mean and confidence interval and the exact P values are listed in the Figure Legends, where appropriate in the main text and within the Figures 1-4 in the main text and Figure Supplements.

(For large datasets, or papers with a very large number of statistical tests, you may upload a single table file with tests, Ns, etc., with reference to sections in the manuscript.)

**Group allocation**

- Indicate how samples were allocated into experimental groups (in the case of clinical studies, please specify allocation to treatment method); if randomization was used, please also state if restricted randomization was applied
- Indicate if masking was used during group allocation, data collection and/or data analysis

Please outline where this information can be found within the submission (e.g., sections or figure legends), or explain why this information doesn’t apply to your submission:

Group allocation was not relevant for our submission.

**Additional data files (“source data”)**

- We encourage you to upload relevant additional data files, such as numerical data that are represented as a graph in a figure, or as a summary table
- Where provided, these should be in the most useful format, and they can be uploaded as “Source data” files linked to a main figure or table
- Include model definition files including the full list of parameters used
- Include code used for data analysis (e.g., R, MatLab)
- Avoid stating that data files are “available upon request”

Please indicate the figures or tables for which source data files have been provided:

Figures in the main text include all the data for bacterial growth rates within the scatter plots, and all the data for qRT-PCR measurements and quantification of lamellar body size, number, and volume. A summary of the code used to calculate growth rates is included in the Materials and Methods. Annotated code used for data analysis written in Matlab, raw data for bacterial fluorescence intensity over time that was used to calculate growth rates, and image stacks related to Fig. 1, Fig. 1 – figure supplement 3 and Fig. 2 – figure supplement 1 will be uploaded to the EPFL community pages at Zenodo prior to publication.
